# Primary Care Clinician Perspectives on Patient Navigation to Improve Postpartum Care for Patients with Low Income

**DOI:** 10.1089/whr.2022.0064

**Published:** 2022-12-15

**Authors:** Abigail Filicko, Kaitlin Huennekens, Ka'Derricka Davis, Brigid M. Dolan, Brittney R. Williams, Joe Feinglass, William A. Grobman, Michelle A. Kominiarek, Lynn M. Yee

**Affiliations:** ^1^ Division of Maternal-Fetal Medicine, Department of Obstetrics and Gynecology, Northwestern University Feinberg School of Medicine, Chicago, Illinois, USA.; ^2^ Department of Family Medicine, Swedish First Hill, Seattle, Washington, USA.; ^3^ Division of General Internal Medicine and Geriatrics, Department of Medicine and Medical Education, Northwestern University Feinberg School of Medicine, Chicago, Illinois, USA.; ^4^ Division of General Internal Medicine and Geriatrics, Department of Medicine and Preventive Medicine, Northwestern University Feinberg School of Medicine, Chicago, Illinois, USA.; ^5^ Division of Maternal-Fetal Medicine, Department of Obstetrics and Gynecology, The Ohio State University College of Medicine, Columbus, Ohio, USA.

**Keywords:** care coordination, patient navigation, postpartum care, transitions of care, primary care, clinician perspectives

## Abstract

**Background:**

Birthing individuals experience significant physical and psychosocial transitions during the postpartum period. Despite amplified health needs, many individuals do not successfully transition from obstetric to primary care. Patient navigation provides a patient-centered solution that has been applied to other health care specialties resulting in improved care coordination and patient engagement for populations in greatest need. Our objective was to understand primary care clinician perspectives regarding the role of navigators in improving postpartum care for individuals with low income.

**Methods:**

In this qualitative investigation, we conducted focus groups with primary care clinicians from family and internal medicine specialties. Semistructured interview guides addressed clinician perceptions of navigator roles during the postpartum period and recommendations for navigator training. Focus group discussions were digitally recorded, transcribed, and analyzed *via* a constant comparative method.

**Results:**

Twenty-eight primary care clinicians, including 26 physicians and 2 advanced practice registered nurses, participated in 8 focus groups. Participants reported favorable attitudes toward implementation of a postpartum patient navigation program. Themes regarding useful navigation services included streamlining obstetric to primary care transition, enhancing visit effectiveness, creating personalized postpartum care, and providing patient- and clinician-focused education. Recommendations for navigator training included education on basic medical concerns that are common in the postpartum period, health information privacy and electronic health record use, health care systems, and community resources.

Clinical Trial Registration number: NCT03922334.

**Conclusions:**

Primary care clinicians were highly receptive to the concept of patient navigation as a process to improve health in the postpartum period through enhanced care coordination and improved patient knowledge, engagement, and self-efficacy.

## Introduction

During the postpartum period, birthing individuals and their families experience significant changes in their health and social roles. While adjusting to new familial roles, physical, mental, and social needs evolve and frequently require close engagement with health care teams.^1,2^ Numerous health agencies and professional societies, including the American College of Obstetricians and Gynecologists, World Health Organization, and the U.S. Preventive Services Task Force, provide guidance on the types and timing of postpartum care services needed to optimize the health of individuals in the first year after giving birth.^3,4^

Despite these increased needs and recommendations, postpartum care remains inconsistent and challenging. A major barrier to postpartum care is the underutilization of postpartum health care services. Postpartum visit attendance rates range between 50% and 63%.^5–7^ Furthermore, social and economic disparities exist in care attendance and quality. Several studies have identified that birthing individuals at greatest risk of missing critical postpartum appointments, laboratory work, and screenings are those who have low income, are from minoritized groups, use publicly funded health insurance, and receive limited prenatal care.^2,6,8–12^ There is also a deficit in the transition from obstetric to primary care, and many birthing individuals do not receive optimal preventive or problem-oriented care in their first year after giving birth.

In other medical specialties, patient navigation has proven to be an effective strategy in improving retention, management, and outcomes for patients while reducing health outcome disparities in marginalized populations.^13–16^ Patient navigation provides individualized assistance and expertise to patients and their families, helping them overcome logistical, financial, educational, and social barriers and enabling timely and comprehensive health care. With the complexity of the health care system, known gaps in postpartum care, and increasing recommendations for more continuous and comprehensive postpartum care services, patient navigation has been proposed as an effective intervention for the postpartum transition period.^17–19^ In a theoretical model for patient navigation, McKenney et al described various mechanisms through which navigators support birthing individuals by enhancing access to care, promoting patient self-efficacy, and sustaining engagement within the health care system.

Within our research group, previous work on patient navigation in the obstetric context has explored obstetric clinician perspectives to identify the specific navigation roles and services that would benefit birthing individuals in the postpartum period.^20^ However, little research has assessed the needs and perspectives of primary care clinicians who are essential to the successful postpartum transition from obstetric to primary care. Thus, we designed this study to build upon our previous work and elucidate primary care clinicians' perspectives on patient navigation for postpartum patients with low income. In addition to uncovering clinicians' views and understanding of the patient navigation concept, we sought to identify their ideas regarding key patient navigator services and necessary training modules for navigators.

## Materials and Methods

This is a qualitative investigation to evaluate health care clinician perspectives on patient navigation during the transition from obstetric to primary care in the postpartum period. Study participants were recruited from various health care settings including a large academic tertiary care center, federally qualified health centers, and private practices throughout Chicago, Illinois. Together, these sites serve a large and diverse population of primary care patients, including patients transitioning from obstetric to primary care. Patients at these sites receive care from advanced practice registered nurses, physician assistants, and physicians from family and internal medicine specialties. These health care clinicians, including trainee physicians, who had experience in managing the care of birthing individuals in the postpartum period were eligible for this study, given the desire to explore a diverse range of perspectives and experiences. All participants were older than 18 years, English-speaking, and provided written consent for participation in the study.

The Institutional Review Board at Northwestern University approved this study, which was conducted as a preparatory component for a randomized controlled trial of postpartum patient navigation.^21^

Focus groups were conducted as 60- to 120-minute interviews with small groups of clinicians. Group numbers varied based on participant availability, and groups were purposefully mixed such that trainee physicians, attending physicians, and advanced practice registered nurses participated together. A semistructured interview guide (Supplementary Data S1) was used to guide discussion and prompt study participants to share experiences in caring for postpartum individuals. The guide was designed by the research team who has previous experience with assessing obstetrics and gynecology clinician perspectives on patient navigation and barriers to care in the postpartum period.^20,22,23^ Given the known health inequities in the postpartum period, our study focused on the experiences and care processes among birthing individuals with low income. Topics discussed included navigator services that would decrease burdens faced by patients with low income and their health care clinicians. In addition, important training topics and experiences for navigators were discussed.

Focus group interviews took place from December 2019 to February 2021. The first three were conducted in-person by two trained researchers. The remaining five focus groups were conducted during the coronavirus pandemic and were facilitated over a videoconferencing platform by two to three researchers. All participants agreed to the recording of focus groups and the subsequent transcription with deidentification of responses. For their time and expertise, participants received $50 gift cards.

Digital recordings of focus groups were professionally transcribed and uploaded to Dedoose (www.dedoose.com), a secure web-based application for qualitative data organization and analysis. The research team conducted qualitative analysis through an iterative coding process consisting of theme identification, modification, and refinement using quotation selection methods advocated by Kurasaki.^24^ The analysis began with two authors reviewing two focus group transcripts through which emerging themes and subthemes were identified and defined. This preliminary codebook was then applied to two additional transcripts that allowed for thematic modification and reclassification. The codebook was subsequently reviewed by the research team to achieve consensus. Themes were clarified, refined, and collapsed through this iterative process. The final codebook was applied to all transcripts and a total of 575 excerpts were coded.

## Results

### Study participants

Twenty-eight health care clinicians participated in a total of eight focus groups, with two to six clinicians in each group (Table 1). The groups included 26 physicians and 2 advanced practice registered nurses, with equal proportions of family and internal medicine specialties represented. Clinical practice experience varied from <1 year (resident) to more than 25 years (faculty). Participants addressed the overarching domains of navigator services and navigator training, discussed in depth below.

**Table 1.** Study Participants (*n* = 28)

Position	Clinician type	*n*
Physicians	Family medicine resident	9
	Internal medicine resident	7
	Public health and preventive medicine fellow	1
	Family medicine faculty	2
	Internal medicine faculty	7
Advanced practice registered nurses	Family medicine advanced practice registered nurse	1
	Family medicine adult nurse practitioner	1

### Navigator services

Study participants shared their definitions of patient navigation and described their prior experiences working with patient navigators. They were then encouraged to identify services they believed patient navigators could perform to address barriers faced by both patients and clinicians in the postpartum period. Themes from these discussions included patient navigator services that *streamline obstetric to primary care transition, enhance visit effectiveness, create personalized postpartum care,* and provide *patient-* and *clinician-focused education* (Tables 2 and 3). Within each of these themes, additional subthemes were identified.

**Table 2.** Primary Care Clinician-Identified Patient Navigation Services for the Postpartum Period

Theme	Subtheme	Exemplary quotation
Streamline obstetric to primary care transition	Medical record sharing	“So for patients you know who deliver in a different hospital system, if the navigator could get those records, that would be great because it's so hard to get records.”
	Appointment management	So an ideal situation for a navigation would be someone who call[s] the patient, helps arrange the actual appointment so the patient is not calling and trying to arrange it when they've got so much else on their plate…”
	Clinician intercommunication	“…making sure that that new provider is aware that this is a patient who is like coming in for transition of care from OB [obstetrics] to primary care…potentially helping facilitate a conversation between the two providers.”
Enhance visit effectiveness	Identify patient's health and social needs	“I think a navigator could help to pinpoint the needs of the patient. Social, medical, emotional, social work wise, and let us know what the key points are that need to be addressed from the patient's perspective. I think that would help us to know what we need to focus on most, and what means the most to the patient.”
	Create a postpartum care checklist	“…it would be helpful if there was kind of a to-do list that …the navigator provided the primary care doctor with…if they notice that something's slipped through the cracks after a year, if the navigator could just kinda nudge physician, maybe just write a little EPIC note saying hey, this patient hasn't received screening for cardiovascular diseases or something. And needs it. I think that might be helpful.”
	Coordinate transportation support	“I've had patients who repeatedly couldn't make their appointments due to transportation issues… [patient navigators] could help find access to safe transportation for these patients.”
	Reinforce clinician instructions	“A big part of our role as… nurse practitioners is education and so I feel that sometimes a person needs to revisit a topic a few times to have truly engrained understanding and so if there is that external kind of thread that's also doing some education, that makes for a more robust visit.”
Create personalized postpartum care	Community member	“I think one of the biggest ways that my health advocate or who is in a way a patient navigator works is that she is a part of the community in which my patients live and so there are some cultural things that she understands in the patients that are really just not something I can understand and so I think that having someone explain it to them in a way that a member of their community understands it is a powerful thing.”
	Easily accessible to patients	“If they [patient] have a person who they feel like they can talk to and reach out to, so having them be aware that… like here's the navigator for your system, please reach out with any questions as they come up and they can help be another contact person…”

**Table 3.** Primary Care Clinician-Identified Education Given by Patient Navigators in the Postpartum Period

Themes	Subthemes	Exemplary quotation
Patient-focused education	Common postpartum concerns	“…the patient navigator might be someone that the patient uses as an initial resource…and so if the patient is describing having like bleeding after her pregnancy the patient navigator has at least some basic understanding of what's normal and what's not could help with triage for that patient and can also serve as like a point of reassurance without necessarily having to go to another doctor's appointment.”
	General health principles	“…if they have some education about how to talk about a healthy diet, and exercise, which is very general health information. I mean that's an added bonus.”
	Importance of primary care transition	“I speak to some patients that have lots of resources but don't understand the division in roles or scope of practice of the OB [obstetrician] versus the primary care person. They think it's perfectly fine just to yearly keep going back to the OB [obstetrician] who doesn't screen for you know basic stuff. So yes, I think the navigator would be great for explaining the roles.”
	Navigating the health care system	“[For] low-income, immigrant women, who may or may not be oriented to the culture of medicine in the U.S…going to the doctor while you're pregnant makes sense, but then going to the doctor for primary care when you're healthy… may not be something that, this idea that you're used to. So in addition to all of like the practical social work, social services referrals… the orientation and education of patients to kind of how the U.S. medical system functions.”
Clinician-focused education	Community resources and services	“…having like a list of resources that they [patient navigators] can refer to for the patient…to know what resources are available within the hospital first, of course, and then within the community to help them.”
	Patient's community	“I think…we can also think of navigators, not only as like resources for the patients toward our systems, but also educating providers about the communities that our patients come from. Because like we said, there are languages that our patients speak that we've never even heard of…So I think, you know seeing patient navigators not only as people to be trained, but people who can help educate us in our health systems about where our patients are coming from, is also like a strengths-based model.”

#### Streamline obstetric to primary care transition

The theme “streamline obstetric to primary care transition” comprised the set of services that navigators can complete to facilitate the transition process between obstetric and primary care clinicians. Subthemes included navigators assisting with medical record sharing, appointment management, and clinician intercommunication (Table 2). Frequently, participants identified timely medical record access as a major barrier to providing care in the postpartum setting. Participants believed navigators could effectively increase medical record access either through faxing records before appointments or ensuring patients brought their medical records to their visits. Appointment management included not only helping to arrange appointments, but also following up with patients to ensure that appointments were attended. Appointment management was seen as a function that not only eased the logistical burdens of transitioning patient care, but also enhanced patient access to care.

As one participant described, navigators can help patients “make appointments and follow recommendations from their healthcare providers…” and assist with “the in-between, the infrastructure-type stuff that patients from a different cultural context or with fewer resources might have disproportionate difficulty with.” Facilitating clinician intercommunication was also identified as a service that could improve the postpartum transition process. Serving as a liaison between patients' primary care clinician, obstetric care clinicians, and other health care team members, navigators are uniquely positioned to connect clinicians and facilitate information sharing in the postpartum transition process.

#### Enhance visit effectiveness

Study participants also identified several navigator services that would *enhance visit effectiveness*. Completed before and after office visits, these services would allow clinicians to optimize health care provision and the use of appointment time. Mechanisms to enhance visit effectiveness include a navigator's ability to identify patients' health and social needs, create a postpartum care checklist, coordinate transportation support, and reinforce clinician instructions (Table 2).

Clinicians often felt limited in their ability to assess the diverse physical, mental, emotional, and social needs of their patients due to the restricted lengths of time for appointments. When meeting a patient for the first time during the postpartum period, these time constraints were especially challenging. To prioritize patient management, counseling, and services, participants envisioned navigators conducting a preliminary assessment of patients' needs and sharing this information with clinicians before appointments. As one participant described,

“a patient navigator…she reached out to me…probably within a week of the new appointment and told me what happened with the delivery, every single concern that the patient had and wanted addressed. And so I had an outline for the appointment myself…[and] the patient also didn't have to remember the 3–4 different things she wanted to bring up.”

In addition to collecting and sharing information on patient needs, participants were enthusiastic about the creation of a postpartum care checklist by navigators. This task would involve navigators organizing a “to-do” list for patients including the referrals, laboratory testing, and health care maintenance needs those patients had been instructed to complete by various clinicians. Particularly useful for postpartum patients with complicated pregnancies, numerous comorbidities, or previously fragmented health care, a postpartum care checklist would serve as a centralized record-keeping of patient care needs. When shared with primary care clinicians, this checklist could facilitate completion of necessary follow-ups at subsequent health care visits. As one study participant summarized: “… [patient navigators] could already have a checklist of things…they can go through the checklist and make sure things have been accomplished and if they're lacking, like the pap smear or flu shot, it can be [completed].”

Participants were highly receptive to receiving reminders from patient navigators to ensure completeness of care in this transitional period where details may commonly be overlooked.

Other important navigator services cited included reliable and timely transportation support and reinforcement of clinician instructions. Collaborating with social work or coordinating local transportation services, navigators could help ensure that patients are physically present for their primary care appointments. In addition, navigators could remind patients of their health goals, clinician counseling, and instructions. By ensuring that patients are able to attend their scheduled appointments and reiterating messages from clinicians, navigators can enhance the productivity of health care visits.

#### Create personalized postpartum care

Study participants identified the theme to *create personalized postpartum care* as a unique strength of the patient navigator role. As lay individuals who work closely with patients in the coordination of their various health services, navigators are easily accessible to patients. Navigators are also frequently from the same communities as patients, adding to their cultural awareness and sensitivity. Clinicians believed these qualities allow navigators to humanize the health care experience, particularly during the challenging postpartum transition period (Table 2). Summarized by one participant, “patient navigation is a way to help personalize the…very impersonal and sort of factory disjointed nature of our health care system.”

#### Patient- and clinician-focused education

Finally, participants viewed patient navigation as a potential strategy to disseminate *patient-* and *clinician-focused education* in the postpartum period. Subthemes of patient-focused education included common postpartum concerns, general health principles, the importance of the primary care transition, and navigating the health care system (Table 3). Although navigators are typically individuals without formal health care training, clinicians considered them a part of the health care team, and as such, potential sources of information for patients. For example, one participant suggested that for patients with gestational diabetes, “a navigator could discuss healthy food options and ways to monitor and alter their diet.” Another saw a navigator's role in vaccine education:

“I know I've come across a lot of patients who are unwilling to vaccinate their children. [Navigators can] discuss the implications on why we vaccinate, how it can benefit their children, and assess why they may have some type of apprehension when it comes to vaccines.”

Beyond acute postpartum and general health needs, navigators can educate patients on the nuances of the U.S. medical system. Particularly useful for immigrant individuals or those with minimal prior health care experiences, navigators can explain the purpose of preventive health services and how different clinicians and specialties interact. As described by one participant, navigators can explain that “…after pregnancy, primary care is about prevention and there are things we do about prevention in the United States that might not have been available or common where you came from before now.” This type of patient education may not be directly addressed through interactions with other health care clinicians or social workers, thus representing a unique opportunity to empower patients to more effectively engage in their health care.

Navigators were viewed as educators for clinicians as well (Table 3). Clinician-focused education topics included resources in patients' communities. Several clinicians reported lacking knowledge of resources and services available for postpartum individuals. Especially in settings where social work access is limited, navigators were viewed as a solution: “I think finding those resources that might be beneficial to patients is probably the biggest thing…if the patient navigator can bring that to the table, that'd be extremely helpful.” Lastly, navigators can bring insight into the community and culture of patients. If navigators share similar backgrounds with their patients, they can serve as a “bridge” between the medical system and patients' local communities. Through this bidirectional connection, navigators can help clinicians better understand the experiences, motivations, and influences of their patients as well as promote comprehensive patient care and cultural humility.

### Navigator training

After detailing the scope of services of postpartum patient navigators, study participants were prompted to discuss their recommendations for navigator training. Navigator training was described as the breadth of education topics and experiences that would adequately prepare navigators to perform their suggested postpartum services. Emerging themes included training on *basic medical concerns, health information privacy and electronic health record (EHR) use, hospital systems,* and *community resources* (Table 4).

**Table 4.** Primary Care Clinician-Identified Training Topics for Patient Navigators

Theme	Exemplary quotation
Basic medical concerns	“I think that they should definitely verse them on like different types of complications that women may experience postpartum. Whether it be like significant blood loss, and you know, maybe a lot of blood clots. Like different things like that. So I think that clinical knowledge is very important for these patient navigators.” “I was thinking in addition, and this would kind of help, so if they had some level of medical knowledge. I find that women often don't know who to ask. Where they might think like alright, am I supposed to ask my OB, am I supposed to ask my pediatrician, am I supposed to ask my internist? So someone that had some kind of medical training that could help coordinate… someone who had a little bit of medical knowledge who could point them in the right direction…”
Health information privacy and electronic health record use	“…at least basic HIPAA training, and the understanding that especially if you're trying to use in-language, in-language health navigators, to speak the languages of your patients, oftentimes these are small immigrant communities, in which people know each other. And therefore privacy concerns, you know if this is your uncle's cousin who knows your business that you know whatever, like the religious institution you're at, that can be a concern for privacy, especially working with lay individuals who aren't used to the same like HIPAA regulations.”
Health care system	“I also feel like training about how the health care systems work, and like how they talk to each other would be really important. Because I feel like that's what a lot of patients have difficulty understanding. And it's, and they have difficulty understanding it because it's not necessarily intuitive.”
Community resources	“If the navigators could have lists of resources that they know…they're part of the community, they know like okay where they can go for counseling groups, just things like that.”

HIPAA, Health Insurance Portability and Accountability Act.

#### Basic medical concerns

Given the expressed desire for navigators to operate as patient educators, participants agreed that navigator training should encompass education on basic medical concerns including common health problems encountered in the postpartum period. A critical element of this training would include how to recognize “warning signs” and direct patients to the appropriate clinicians. Described by one participant,

“I think providing at least like basic information about why we are concerned about this time period in particular and what is high risk about this period, whether it is complications related to conditions that arose during pregnancy or chronic conditions…understanding what it is that we as providers are concerned about would be helpful, I think.”

Another added,

“I think a patient navigator is seen as a touchpoint to the health care system. And so, whether they like it or not, they might have some medical questions directed towards them. And so it's important to make sure that they at least are able to triage those questions effectively.”

#### Health information privacy and EHR use

Training on the Health Insurance Portability and Accountability Act (HIPAA) and utilization of the EHR should be a component of navigator training. Understanding patient privacy rights and navigating the EHR are essential to many navigator services, including sharing medical records with primary care clinicians. In addition, many participants envisioned navigators utilizing the EHR to share important patient updates. One stated: “…It may be nice if the patient navigators also are able to chart or just do like a quick note… I think that's just a way to kind of keep track of what they'd been working on together.” Understanding how to share patient information ethically and effectively would allow navigators to complete these services.

#### Hospital systems

Participants felt strongly that navigator training should also include information on the operations of the health care system. Described as the “hidden curriculum” of medical training, understanding health care from a systems level would allow navigators to best assist the care coordination of their patients. This would include knowledge of health insurance, interactions between hospital systems and departments, and the roles of various health care team members. In addition, more granular systems training on topics such as the physical layout of clinical settings and “…the nuances of scheduling for the clinicians you are trying to get your patients to” could further enhance navigator efficacy.

#### Community resources

Finally, our analysis revealed that awareness of relevant community resources would be a critical component of training. Participants envisioned navigators' primary role as “help[ing] [patients] kind of connect to other services even outside of their medical visit or medical needs… So transportation, childcare, or nutrition.” Navigators would need to be informed on the social services available within a local community, and importantly, how patients may access these services within the postpartum period.

## Discussion

The postpartum period is a pivotal time in the health and well-being of birthing individuals and their families. Although it is a challenging period for many, individuals with low income face additional barriers in acquiring critical health and social services.^22,25^ Patient navigation in the obstetrical setting is a potential patient-centered solution to barriers encountered during prenatal and postnatal care.^17,18,26,27^

In prior work, patients viewed navigation services positively and had improved health outcomes with navigator support.^18,23^ With growing evidence that patient navigation services may be beneficial in the postpartum period, feedback on navigation services and training from key stakeholders in postpartum health care can help to facilitate effective implementation of navigation services. Previous work from our research group has assessed perspectives from obstetrics and gynecology-trained clinicians on postpartum patient navigation.^20^ This study explores insights from primary care clinicians who are important players in achieving postpartum care transitions for individuals with low income.

**FIG. 1.**
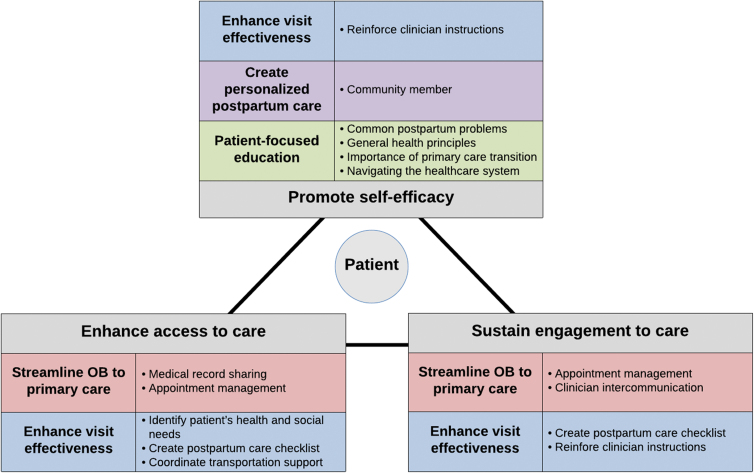
Model of patient navigator services. An adaptation of McKenney et al's model for patient navigation, highlighting the primary care clinician-identified navigator services and their effects on postpartum patients.

In 2018, McKenney et al considered the utilization of patient navigation in obstetric and gynecologic settings and proposed a model by which patient navigation roles and services can promote patient self-efficacy, enhance access to care, and sustain engagement with care. In our investigation of primary care clinician perspectives on postpartum patient navigation, we identified several navigation services (*e.g.*, sharing medical records, facilitating appointment management, aiding clinician intercommunication, coordinating transportation support, and providing patient-focused education) that support McKenney's model (Fig. 1). Moreover, our analysis identifies additional services that expand upon this three-pillar model. As a member of a patient's local community, navigators are uniquely positioned to provide psychosocial support to patients, ultimately promoting their self-efficacy. Identification of a patient's health and social needs and creation of a postpartum care checklist facilitate more productive health care interactions, bolstering access to care and sustained engagement with care.

Essential to successful patient navigation is comprehensive navigator training. As identified in our study, patient navigators must develop a diverse repertoire of knowledge, skills, and experiences for optimal functioning. One way to achieve this is through the development and implementation of a formal patient navigator training system. While generating a novel patient navigation program for publicly insured individuals in the Navigating New Motherhood 2 trial, Yee et al developed a specialized navigator curriculum.^19^ This training focuses on six elements (principles of patient navigation, knowledge of prenatal and postpartum care, health education and health promotion principles, cultural sensitivity and health equity, care coordination and community resources, and EHR systems) explored through formal didactic sessions as well as observational and experiential learning. This framework for navigator training is well supported by the recommendations from primary care clinicians in this study.

In addition, our findings emphasize the importance of health care system awareness for navigators. This aspect of training includes sufficient understanding of the hospital setting, clinician roles, and logistical procedures for clinic- and hospital-based care. Using a specialized postpartum patient navigator training program as proposed by Yee et al, with emphasis on hospital system operations, can allow for lay individuals with limited clinical or social work experience to operate successfully in the patient navigator role. As increasing nursing and social service shortages continue to impact health care provision throughout the country, the ability to comprehensively train lay navigators to effectively support postpartum patients is a great advantage of this patient navigator model.

The training and service recommendations identified in this study can ensure that these navigation programs are optimized to provide the greatest benefit for patients and clinicians. Enhanced emphasis on health care system training for navigators can supplement existing educational experiences and better equip navigators to support patients. Focusing navigator efforts on services that streamline the obstetric to primary care transition and enhance visit effectiveness can standardize navigator workflow, thereby achieving the goal of improving health after pregnancy. Future investigations must examine patient health outcomes associated with navigator services as well as cost-effectiveness of navigation programs. In addition, evaluating navigation needs between teaching and nonteaching settings will also be advantageous, particularly since recommendations from trainee versus nontrainee clinicians may vary due to different experiences with system-level care coordination, local community resources, and priorities.

Finally, in an effort to promote the scalability and sustainability of navigation services, future studies can explore the benefits of virtual navigation.

The strengths of this study include its in-depth analysis of primary care clinician needs and perspectives while managing the care of postpartum individuals. Despite being key members of the postpartum care team, primary care clinicians have been underutilized in the development of obstetric care coordination programs and have unique insights into the challenges that patients and clinicians face in the year following childbirth. Another strength is the specificity in which clinicians were able to identify the exact services needed and how navigators could accomplish these tasks. Direct feedback on needs and solutions can allow the findings from this study to be easily incorporated in the existing or future patient navigation systems.

Limitations were also present in this study. Perspectives were only elicited from clinicians within one Midwest city. In addition, most participants reported that <10% of their practice comprised postpartum patients. However, we do not perceive this to be a significant weakness because this practice pattern reflects the typical composition of primary care patients. Furthermore, many themes identified in this study mirror similar studies evaluating patient navigation in obstetrical and oncologic specialties.^16,20,28–30^ Lastly, because this study focused on the perspectives of clinicians, critical insights from patients and patient navigators on beneficial navigator services and training must be synthesized in future work.

## Conclusions

In the pursuit of improving the health of birthing individuals, this study shares insights from primary care clinicians on the unique benefits, services, and training aspects of patient navigation programs. Supplementing the care provided by health care personnel, patient navigators can provide numerous services that directly address the barriers faced by individuals with low income and their primary care clinicians. Together, these findings will contribute to the growing literature exploring patient navigation as one solution to the countless disparities that exist in postpartum care for individuals in greatest need.

## Supplementary Material

Supplemental data

## Abbreviations Used

EHR: electronic health record
HIPAA: Health Insurance Portability and Accountability Act
