# Supplementary material for: Primary Care Clinician Perspectives on Patient Navigation to Improve Postpartum Care for Patients with Low Income
Source: Womens Health Rep (New Rochelle). 2022 Dec 15;3(1):1006–15. doi: 10.1089/whr.2022.0064 (PMC9811840; doi:10.1089/whr.2022.0064)
Supplement: Supplemental data [file Suppl_DataS1.docx]

**Supplementary Data S1. Semi-structured interview guide for primary care provider focus groups on patient navigation in the postpartum setting**

**Part 1: Clinical background and experiences**

- What is your role and how long have you been working in this position?
- What is your best estimate of the proportion of your patients who are postpartum? How often do you typically recommend seeing them?
- In your experience, what are some of the biggest challenges for your patients during pregnancy and after birth? For example, can you describe challenges related to access to or engagement in care?
  - Why are they challenges?
  - What resources are still lacking for this patient population?
- What outside resources do you recommend patients use during the postpartum period, if any?
  - Which resources do you find are most helpful for patients?
  - What makes them especially helpful?
  - In a perfect world, what additional resources would you like to be able to offer patients during the postpartum period?
- What do you think is the most effective method for engaging with patients outside of the clinical setting and why? This could be for scheduling, health education, clinical concerns, or other needs. For example, MyChart, email, phone, or others.
- When you see a new patient who is postpartum or a return patient who is seeing you after she has given birth, do you receive any communication from her obstetric team about her obstetric course and health?
  - [possible probe] For example, do you ever receive communication via Epic, a written summary, or other direct communication from her obstetrician?
  - [probe for when there is a transition process] What aspects of the obstetric to primary care transition run smoothly in your practice?
- What are some of the clinical challenges you face as a provider for this population?
  - For example, do you receive adequate records from the obstetrical team?
    - Does this differ based on whether the patient delivered at this institution versus elsewhere?
  - Where do you primarily look for records when using Epic – for example, do you prefer to use the problem list or the clinical notes? Keep in mind obstetric patients may not have hospitalization discharge summaries if their birth was uncomplicated.
  - Do you feel you have a full understanding of the delivery and early postpartum events?
- Describe an ideal “hand off” from obstetric care to primary care.
- Tell me about problems with providing care during this transition.
  - What non-clinical tasks do you find yourself doing to support low-income postpartum women?
  - Who are key members of your team who provide care with you for these patients?
    - [possible probe] For example, social work, nurses, or nurse practitioners.

**Part 2: Navigation**

- Could you define patient navigation in your own terms?
  - Do you have prior experience working with patient navigators?
- When you think of patient navigation, what specific services or processes would you envision being most helpful to patients transitioning from obstetric care to primary care?
- Keeping in mind that most patient navigators are lay individuals without professional health care training, please think about what areas – either clinical or administrative – you think are most essential for training a postpartum patient navigator.
- What can the navigator do to ease the administrative, educational, or logistical burdens of your role?
- Can you describe other ways in which patient navigation can help you?
- How often and by what means would you like to communicate with the navigators?

**Part 3: Navigation outcomes**

- Tell us how you think the navigator can support patients and clinic staff in achieving the following health and healthcare outcomes.
  - Preventive care – vaccines, screening tests, etc
  - Diabetes testing for women with GDM
  - Screening for atherosclerotic cardiovascular diseases for women with preeclampsia, hypertension, small-for-gestational age neonates, and preterm birth
  - Gestational weight loss
  - Transition to and retention in primary care
